# Redefining power in social psychology

**DOI:** 10.1111/bjso.70031

**Published:** 2025-12-12

**Authors:** Karim Bettache, Giovanni A. Travaglino, Peter Beattie

**Affiliations:** ^1^ Faculty of Social Science The Chinese University of Hong Kong Hong Kong China; ^2^ Royal Holloway The University of London Egham UK

**Keywords:** intergroup relations, power, resistance, social dominance, WEIRD

## Abstract

This article synthesizes cutting‐edge research from our special issue examining power across groups, communities and nations to advance a fundamental reconceptualization that reveals power's inherent plurality, dynamism and cultural embeddedness. Drawing on evidence from a diverse set of countries and innovative methodologies, we demonstrate that traditional definitions of power as asymmetric resource control fail to capture its real‐world complexity. The collected research reveals three critical insights. First, power operates through multiple, intersecting mechanisms—from ideological frameworks like hegemonic masculinity to seemingly benevolent helping behaviours that maintain hierarchies. Second, power's meaning and operation vary dramatically across cultural contexts, with the power‐status relationship ranging from nearly synonymous in some societies to entirely disconnected in others. Third, marginalized groups develop sophisticated alternative conceptualizations of power as collective resistance, cultural preservation and mutual aid that enable transformation despite structural disadvantages. These findings necessitate theoretical frameworks that accommodate power's plurality while identifying general principles, examining dynamic processes rather than static attributes and integrating multiple levels of analysis. We argue for moving beyond imposed definitions to understand how diverse groups conceptualize their own agency, examining not just domination but resistance and developing interventions that promote more equitable power distributions.

Power is the often‐invisible architecture of human social life. It shapes every interaction, from the most intimate personal relationships to the grand theatre of geopolitics (Fiske & Berdahl, 2007; Guinote & Vescio, 2010). Power determines whose voices are heard in boardrooms, classrooms and the media (Keltner et al., 2003), whose perspectives, interests and beliefs shape public policy (Lukes, 1974, 2005), and whose lives are valued or discarded (Sidanius & Pratto, 1999). Because it underpins the capacity to exercise, or to restrain, violence, power is central to the formation of political authority and the legitimacy of social order (Gaventa, 1982; Weber, 1978). It enables cooperation, structures the management of conflict and governs how wrongdoing is sanctioned (Tyler, 2006). It manifests in the subtle dynamics of everyday conversations—who interrupts whom, who takes up space, who defers (Ridgeway, 2011)—and in the dramatic confrontations between protesters and police, between colonizers and the colonized, between those who would preserve hierarchies and those who would dismantle them (Drury & Reicher, 2009). Understanding power, therefore, is not merely an academic exercise but an urgent necessity for anyone seeking to comprehend the profound injustices and possibilities for remediation that define our contemporary moment (Martín‐Baró, 1994).

The current special issue arrives at a critical juncture in human history. Around the globe, we are witnessing tectonic shifts in power dynamics that demand new theoretical frameworks and empirical approaches. The COVID‐19 pandemic laid bare the deadly consequences of power inequalities, as marginalized communities suffered disproportionate mortality while wealthy nations hoarded vaccines (Bambra et al., 2020; Shadmi et al., 2020). The rise of authoritarian populism across democracies once thought stable has revealed the fragility of liberal institutions and the enduring appeal of strongman politics (Bettache & Chiu, 2018; Osborne et al., 2023). Social movements— from Black Lives Matter to #MeToo, from indigenous rights campaigns to the global protests against genocide in Gaza—have exposed how power operates through intersecting systems of oppression while demonstrating the potential for collective resistance to transform societies (Drury & Reicher, 2000; Van Zomeren et al., 2011, 2012). Meanwhile, the accelerating climate crisis reveals power's temporal dimensions, as decisions made by today's powerful—primarily in wealthy nations and corporations—condemn future generations to inherit a damaged planet at best and ecological catastrophe plus societal collapse at worst (IPCC, 2023; Milfont & Sibley, 2016; Uenal et al., 2021).

These contemporary challenges underscore fundamental questions about power that social psychology must address. How do individuals and groups acquire, maintain and lose power? How does power shape cognition, emotion and behaviour—not just for those who wield it but for those subjected to it? How do cultural contexts and historical legacies influence the meaning and operation of power? How can those without traditional forms of power mobilize collective resources to challenge entrenched hierarchies? And perhaps most crucially, how can psychological science contribute to creating more equitable distributions of power that enhance rather than diminish human flourishing?

## THE PROMISE AND LIMITATIONS OF TRADITIONAL APPROACHES

Social psychology has grappled with these questions for decades, producing invaluable insights while also revealing significant limitations in how the field has conceptualized and studied power. The dominant approach has defined power as *asymmetric control over valued resources*—specifically, the capacity to influence others by controlling outcomes they value and the corresponding freedom from others' influence (Fiske & Berdahl, 2007; Keltner et al., 2003; Magee & Galinsky, 2008). This encompasses control over material resources (money, food, shelter), social resources (status, affiliation, support) and informational resources (knowledge, expertise, access). This resource‐control paradigm has generated robust findings about how power affects individual psychology—enhancing approach tendencies, reducing inhibition, decreasing perspective‐taking and increasing goal‐directed behaviour (e.g. Anderson et al., 2012; Galinsky et al., 2003). Laboratory experiments have demonstrated that even minimal manipulations of power, such as recalling past experiences of having or lacking power, can profoundly influence cognition and behaviour.

Yet this traditional approach has increasingly been recognized as insufficient for capturing power's full complexity and real‐world operation (e.g. Turner, 2005). The focus on resource control and interpersonal influence, typically studied through brief laboratory manipulations with Western undergraduate samples, fails to address several critical aspects of power.

First, it subordinates the role of group dynamics to competition over materials and social resources. However, this overlooks the fact that the importance of such resources is defined precisely in the context of our relationships with our groups (Turner, 2005). For communities that have been systematically excluded from traditional power structures, power may be understood not only as control over things or people but rediscovered as collective resistance, cultural preservation, spiritual connection, or mutual aid (Bettache, 2025a, 2025b; Prilleltensky, 2008). Black Americans, for instance, may locate power in their capacity for collective resilience in the face of centuries of oppression, in the strength of community bonds that have sustained them through adversity, or in their ability to create transformative cultural movements that reshape society (e.g. Twali et al., 2023). Indigenous peoples might understand power through their relationship with ancestral lands, their maintenance of traditional knowledge systems, or their capacity to assert sovereignty despite colonial domination (Adams & Salter, 2007). By imposing a singular, Western‐derived definition of power as resource control, traditional research has paid comparatively less attention to alternative dynamics emphasizing individuals' bonds, connections and their sense of collective identity. These dynamics are equally or more relevant for understanding how counter‐power, or the power to resist, operates in diverse human communities.

Second, the individualistic focus of much power research has obscured how power operates through collective processes, institutional structures and cultural narratives. While laboratory studies can illuminate how individuals respond to temporary shifts in power, they cannot capture how media representations shape public understanding of complex issues like modern slavery or neocolonialism, how neoliberal ideologies structure perceptions of fairness and deservingness, or how colonial histories continue to influence contemporary power relations through internalized hierarchies and ongoing structural violence (Adams et al., 2019; Beattie et al., 2019; Bettache, 2020; Bettache et al., 2020; Bettache & Chiu, 2019a, 2019b). Power is not merely a property of individuals or dyadic relationships but is embedded in social systems, cultural meanings and historical trajectories that require analysis at multiple levels (Travaglino & Abrams, 2019).

Third, the overwhelming reliance on Western, Educated, Industrialized, Rich, Democratic (WEIRD) populations (Henrich et al., 2010a, 2010b) has produced a distorted and culturally limited understanding of power dynamics. The assumption that findings from American or European university students can be universalized to all human societies implies an ontology of merely complicated, not complex, systems and ignores the profound ways that cultural values, social structures and historical experiences shape power's meaning and operation. Research reveals substantial variation in fundamental aspects of power relations across cultures (Bettache & Chiu, 2018, 2019a, 2019b; Gelfand et al., 2011; Hamamura et al., 2018; Torelli & Shavitt, 2010).

Fourth, traditional approaches have often treated power as a static attribute—something individuals or groups simply possess or lack—rather than as a dynamic, contested and performative phenomenon. This static view cannot explain the fluid nature of power in real‐world contexts: how displays of force by authorities can backfire by undermining legitimacy and mobilizing resistance (Drury & Reicher, 2000), how seemingly powerless groups can suddenly coalesce into movements that topple regimes, or how power relations can be transformed through shifts in narrative framing or collective consciousness. Power is not simply held but must be continually enacted, negotiated and defended against challenges.

Fifth, the field has inadequately theorized the relationships between different forms and levels of power. Traditional research has tended to study power in isolated domains—interpersonal power in social psychology, political power in political science and economic power in sociology—without examining how these different manifestations of power intersect and mutually constitute each other. How does micro‐level interpersonal power relate to macro‐level structural power? How do psychological experiences of empowerment or disempowerment connect to objective positions in social hierarchies? How do different bases of power—coercive, economic, ideological and cultural—interact to produce complex systems of domination and resistance? These questions require theoretical frameworks that can bridge levels of analysis and disciplinary boundaries.

Finally, methodological constraints have limited the ecological validity and real‐world relevance of much powerful research. Laboratory experiments, while providing crucial causal evidence, typically involve brief, artificial manipulations that cannot capture the lived experience of those in chronic positions of advantage or disadvantage. The predominance of quantitative methods has decontextualized psychological science and marginalized rich qualitative insights into how people make meaning of power in their daily lives, how they navigate complex power dynamics in natural settings and how they develop strategies of resistance or accommodation (Adams et al., 2019; Bettache, 2020). Moreover, the traditional researcher‐subject power dynamic in psychological research has often reproduced the very hierarchies that power research should interrogate, with marginalized communities studied as objects rather than engaged as partners in knowledge production.

These limitations reflect psychology's historical development within Western, particularly North American, academic institutions that have often served elite interests (Martín‐Baró, 1994). ‘Liberation psychology’, emerging from Latin American contexts marked by extreme inequality and political oppression, offers a crucial counterpoint by centring the experiences and knowledge of marginalized communities, examining how psychological processes maintain or challenge oppressive systems, and explicitly orienting research towards social transformation (Martín‐Baró, 1994; Montero & Sonn, 2009; Watkins & Shulman, 2008). This tradition recognizes that oppressed groups are not merely subjects to be studied but possess sophisticated understandings of power dynamics born from lived experience—what Freire (1970) termed ‘conscientization’, the process through which marginalized people develop critical consciousness of their social reality and capacity for transformative action. Liberation psychology's insights prove particularly relevant for this special issue's examination of power. Its emphasis on ‘critical realism’—acknowledging both material structures of oppression and the psychological processes through which they are maintained or resisted—helps bridge macro‐level analyses of inequality with micro‐level studies of individual experience (Montero & Sonn, 2009).

Several papers in this special issue reflect these core commitments. Haughton et al.'s (2025) discourse analysis reveals how dominant narratives obscure exploitation while appearing to champion victims—precisely the kind of ideological mystification liberation psychology seeks to expose. Solano‐Silva et al.'s (2025) research on how neoliberal ideology leads oppressed groups to view unjust inequalities as fair exemplifies what Martín‐Baró (1994) called the ‘internalization of oppression’. And Moon et al.'s (2025) focus on collective efficacy as enabling communities to resist criminal organizations aligns with liberation psychology's emphasis on collective empowerment through critical consciousness and organized action. By foregrounding these connections, we position this special issue within a tradition that has long recognized power's complexity while maintaining explicit commitment to its transformation.

## TOWARDS A MULTI‐DIMENSIONAL UNDERSTANDING OF POWER

Before examining how this special issue advances our understanding, we must clarify what we mean by ‘redefining’ power. We are not rejecting resource control as irrelevant to what power means but rather arguing it represents only one dimension of a more complex phenomenon. Drawing on Lukes' (1974, 2005) seminal distinction between visible, hidden and invisible forms of power, and Gaventa's (1982) analysis of powerlessness as more than simply lacking resources, we propose that power operates simultaneously through multiple dimensions that cannot be reduced to one another.

***Visible power*** concerns observable decision‐making and conflict—who prevails when preferences clash openly. This dimension aligns most closely with traditional social psychological definitions of power as rooted in asymmetric resource control. The papers in this issue by Wildschut and Insko (2025) and Bartholomaeus et al. (2025) primarily examine this dimension, testing how variations in fate control and reflexive control influence competitive behaviour and developing measures to capture experiences of gaining or losing such control.

***Hidden power*** operates by controlling what issues reach public consciousness and which alternatives are considered (Bachrach & Baratz, 1962). This dimension encompasses agenda‐setting, framing and the selective amplification or suppression of grievances. The papers by Haughton et al. (2025) on media representations of modern slavery and Avery et al. (2025) on how hegemonic masculinity shapes environmental discourse exemplify research on this dimension. Here, power manifests not in direct resource control but in shaping collective attention and the boundaries of legitimate debate.

***Invisible power*** works through shaping consciousness itself—influencing what people see as natural, inevitable or desirable such that they accept or even actively support arrangements contrary to their interests (Lukes, 2005). Solano‐Silva et al.'s (2025) research on how neoliberal ideology leads disadvantaged groups to perceive unjust inequalities as fair outcomes demonstrates this dimension. So does Knab et al.'s (2025) finding that dependency‐oriented helping maintains hierarchies while appearing benevolent, and Gvirtz et al.'s (2025) evidence that high‐SDO individuals punish dominant‐group members who express remorse for harming subordinates—treating ideological transgression as worse than material harm.

Critically, these dimensions are not alternative definitions of the same construct but analytically distinct phenomena that can diverge or align depending on context. A group may possess substantial resources (visible power) yet lack the ability to set agendas (hidden power) or face internalized oppression that prevents mobilization (invisible power). Conversely, as Jeong et al. (2025) demonstrate, groups with limited traditional resources may possess alternative strengths—collective resilience, solidarity, cultural capital—that enable resistance when consciousness shifts.

This multi‐dimensional framework helps clarify what might initially seem as tensions across the papers in this special issue. All examine power, but they focus on different dimensions or their intersections. Some investigate resource control (visible power), while others examine ideological processes (invisible power) or discursive struggles (hidden power). The diversity reflects power's genuine complexity. Our task is not to choose one dimension as the ‘true’ definition but to theorize their relationships and develop methods appropriate to each.

## THE STRUCTURE OF THE CURRENT ISSUE

The collected papers are organized into four sections that reflect distinct but interconnected aspects of power. While any organizational scheme necessarily simplifies the rich connections across papers, we have grouped them to highlight conceptual coherences. While several papers could reasonably fit in multiple sections, our choices aim to scaffold readers' engagement with the full complexity that power encompasses.

### Section 1: Power's psychological dimensions and dynamics

The first section examines power at individual and interpersonal levels, investigating the psychological processes through which power shapes cognition, emotion and behaviour. These papers remind us that while power operates through macro‐level structures and ideologies, it is ultimately enacted through micro‐level psychological experiences and interactions.

Bartholomaeus et al. (2025) address a critical methodological gap that has hampered experimental power research: the lack of validated measures for empowerment—the transitional experience of gaining or losing power. Their development and validation of the Episodic Empowerment Scale (EES) through four rigorous studies provide researchers with a tool specifically designed to capture how people feel in moments when their power shifts, which is precisely what experimental manipulations aim to create.

The key innovation lies in recognizing that empowerment is conceptually distinct from both trait power (one's habitual sense of power across situations) and state power (one's current level of power). Empowerment captures the dynamic experience of change—losing control in one moment, gaining influence in another—that characterizes how people actually experience power shifts in daily life and experimental settings. The authors define empowerment as a transitory state where individuals feel they can influence and be free from the influence of others, with the crucial element being the fundamental shift in perceived power.

Beyond creating the scale itself, the authors propose a novel framework for validating manipulation checks in experimental psychology, establishing four criteria: stable nomological net (consistent relationships with other constructs across conditions), stable internal consistency reliability, sensitivity to the target construct and invariant factor structure across psychological states. The EES meets these criteria while demonstrating superior sensitivity to power manipulations compared to existing measures like the Personal Sense of Power Scale (Anderson et al., 2012). Notably, their work reveals the complexity of isolating power from affect in experimental settings—a challenge that speaks to the intertwined nature of these psychological experiences rather than measurement failure.

This methodological advance enables more precise experimental research on power dynamics. The brief five‐item scale can be seamlessly integrated into experiments, ecological momentary assessments or any context requiring measurement of transitional power states. By providing both a validated tool and a framework for developing manipulation checks, this research strengthens the methodological foundation necessary for advancing our understanding of how power operates across diverse contexts and populations.

Wildschut and Insko's (2025) experimental investigation examines power in the domain of intergroup competition, addressing how power differentials influence the well‐established finding that groups compete more than individuals (the discontinuity effect). Using carefully designed matrix games, they manipulate not just the level of power but also its type—fate control (control over others' outcomes) versus reflexive control (control over one's own outcomes). The results reveal that both forms of power increase competitiveness, challenging theories that sharply distinguish between social and personal power.

The research also demonstrates that groups remain more competitive than individuals even under power asymmetries, suggesting that group contexts fundamentally alter power dynamics in ways that cannot be reduced to individual psychology. Importantly, they replicate prior findings that unequal‐power settings increase competitiveness and find in addition that this effect is driven entirely by high‐power individuals and groups. The finding that greed rather than fear primarily drives competition in these contexts challenges defensive explanations of intergroup conflict and complements recent research highlighting how capitalist contexts shape human behaviour in previously unexamined ways (e.g. Bettache, 2025a; Kasser, 2003). Together, these results suggest that power operates differently at group versus individual levels, with group contexts amplifying competitive orientations regardless of power distributions.

Gobel et al.' (2025) research on power construal provides an elegant capstone by demonstrating that power's effects depend fundamentally on how people understand its origins and meaning. Through surveys and experiments across Japan and the United States—cultures with different power traditions—they distinguish between viewing one's power as achieved through personal merit versus entrusted by others. This distinction proves crucial for predicting whether power leads to prosocial or antisocial behaviour.

When people construe their power as entrusted by others—given by communities, inherited from ancestors, or delegated by constituents—they show enhanced perspective‐taking, greater empathy and more altruistic behaviour. They see power as carrying obligations to those who granted it. When people construe their power as achieved through personal merit—earned through competition, built through individual effort or won through superiority, as in the neoliberal conception of capitalist democracy as a meritocratic political‐economic system—they show reduced concern for others and more self‐serving behaviour. They see power as a personal possession to be used for personal ends.

These effects emerge across cultures despite different baseline rates of each construal, suggesting fundamental psychological processes while leaving room for cultural variation. The research challenges monolithic views of power as inevitably corrupting by showing that corruption depends on meaning‐making. It also suggests practical interventions: reminding powerholders of their interdependence and obligations can promote more responsible use of power. More broadly, it demonstrates that changing how people think about power can change how they use it, opening possibilities for cultural and institutional reforms that promote prosocial power construals.

These papers establish that power's psychological effects depend fundamentally on how people experience and understand it—whether as a resource gained or lost, a basis for competition or cooperation, an entrusted responsibility or personal achievement. But individual and interpersonal psychology unfolds within broader ideological contexts that shape what power means and how it operates, leading us to our second section.

### Section 2: Power, ideology and consciousness

The second section examines what Lukes (2005) identified as power's ‘third dimension’—its operation through shaping consciousness, preferences and the very categories through which people interpret reality. These papers reveal how power maintains itself not primarily through coercion or even visible resource control, but through ideological frameworks that make existing arrangements appear natural, inevitable or just.

Avery et al.' (2025) investigation of how patriarchal ideology shapes gendered responses to environmental change. Their research across two studies (the United Kingdom and Francophone Switzerland) reveals that men's consistently lower pro‐environmentalism compared to women stems not simply from dominance orientations but fundamentally from their higher endorsement of hegemonic masculinity—a culturally shared belief system that legitimizes male dominance through norms of status‐seeking, emotional toughness and rejection of femininity. The authors demonstrate that hegemonic masculinity endorsement creates a worldview in which pro‐environmental change is perceived as threatening because it challenges the patriarchal order that privileges masculine values of control and hierarchy.

This research makes several crucial contributions. First, it identifies hegemonic masculinity as a key ideological framework explaining gender differences in environmentalism, showing that this patriarchal ideology operates through threat perceptions: men who strongly endorse hegemonic masculinity perceive pro‐environmental policies as more threatening, leading to greater rejection of such policies. Second, it reveals that while individuals' social dominance orientation (SDO) is subsumed under their endorsement of a patriarchal ideology (the effect of SDO on threat disappears when hegemonic masculinity is included), ecological dominance orientation represents a distinct belief system about human–nature hierarchies. Third, the research demonstrates how power operates through gendered ideologies to shape responses to existential challenges like climate change, suggesting that addressing environmental crises requires confronting the ideological antecedents of climate policy opinions, including hegemonic masculinity and ecological dominance (and, potentially, providing ‘masculine’ climate policy frames, focusing on *transforming* the economy and *building* renewable energy infrastructure). The finding that hegemonic masculinity endorsement accounts for unique variance beyond dominance orientations reveals how deeply gender ideology is implicated in environmental attitudes.

The article by Gvirtz et al. (2025) examines a puzzling phenomenon at the intersection of power, morality and ideology: how social dominance orientation shapes reactions to hate crime perpetrators' expressions of remorse. Across four experiments using different perpetrator‐victim combinations (anti‐gay hate crimes, police shootings of unarmed Black men, violence against refugees), they find a counterintuitive pattern.

While common sense might suggest that everyone would view remorse as mitigating punishment, the research reveals sharp ideological divisions. Individuals low in SDO—who prefer equality over hierarchy—do indeed recommend more lenient punishment when perpetrators express genuine remorse for harming subordinate group members. For them, remorse signals the possibility of redemption, reconciliation and social repair. But individuals high in SDO—who prefer maintaining group hierarchies—actually recommend harsher punishment when perpetrators express remorse. This effect holds even after controlling for prejudice towards the victim groups, suggesting it reflects something deeper than simple outgroup antipathy.

The authors' interpretation through social dominance theory is compelling: for those who support hierarchical social arrangements, a dominant group member expressing remorse for harming subordinates represents a betrayal of the natural order. It suggests that hierarchies are wrong, that subordinates deserve equal treatment, and that dominators should be accountable for maintaining inequality. This ideological transgression may be even more threatening than the original harm because it undermines the legitimizing myths that justify hierarchy. The findings reveal how power ideologies shape moral judgments in ways that maintain or challenge existing structures, with implications for criminal justice, restorative justice and intergroup reconciliation efforts.

Farkhari et al.' (2025) ambitious cross‐cultural study of right‐wing authoritarianism and threat perceptions illuminates how individual ideologies interact with societal contexts to shape intergroup relations. Drawing on three studies culminating in data from 41 countries with over 52,000 daily assessments, they examine how authoritarianism—the desire for order, tradition and strong leadership—influences perceptions that minoritized groups pose threats to society.

The basic finding that RWA predicts threat perceptions is robust across contexts, with meta‐analytic effects ranging from large to very large. People high in RWA consistently see refugees, immigrants, ethnic minorities and other marginalized groups as threatening social cohesion, cultural values and security. This supports the dual‐process model's proposition that authoritarians view the world as dangerous and see outgroups as sources of threat.

But the crucial contribution lies in examining cross‐level interactions. The relationship between individual RWA and threat perceptions is weaker in countries with high religiosity or high marginalization. The authors interpret this as evidence that authoritarians feel more secure when societal structures already align with their preferences. In highly religious societies where traditional values dominate, or in marginalized societies where strong leadership is normalized, authoritarians may feel less need to perceive threats because the social order already reflects their ideals. This reveals how individual psychology cannot be understood outside its structural context—the same authoritarian orientation produces different outcomes depending on whether surrounding institutions affirm or challenge authoritarian values.

The inclusion of daily diary data alongside cross‐sectional measures provides insight into how threat perceptions fluctuate in daily life. While the patterns are similar, effect sizes are smaller for daily perceptions, suggesting that immediate contexts moderate ideological influences. This methodological innovation opens new avenues for understanding how power‐related ideologies translate into lived experience across different temporal scales.

Solano‐Silva et al.' (2025) analysis of justice perceptions across Latin America provides a regional deep dive that complements the global scope of Wasiel et al.'s (2025) analysis. Using Latinobarómetro data from over 20,000 respondents across 17 countries, they examine how social status and neoliberal contexts shape perceptions of whether societies distribute resources and opportunities fairly. Their conceptualization of social justice goes beyond income inequality to encompass access to education, health care and fair treatment by institutions—domains that capture the multidimensional nature of justice in societies marked by extreme inequalities.

The findings reveal a paradox of neoliberal ideology. While higher‐status individuals predictably perceive greater social justice, the gap between high‐ and low‐status groups is smaller in more neoliberal societies. This is not because neoliberal policies create more actual equality—Latin America remains the world's most unequal region (Clavijo et al., 2021; ECLAC, 2021)—but because neoliberal ideology leads even disadvantaged groups to see inequalities as fair outcomes of meritocratic processes (Bettache et al., 2020). The individualistic emphasis on personal responsibility obscures the role of structural barriers, while the veneration of market mechanisms makes unequal outcomes seem natural and inevitable (Girerd & Bonnot, 2020).

This research demonstrates how ideological power operates to maintain structural inequalities by shaping the very criteria by which people evaluate fairness. It also reveals the political consequences: perceiving greater social justice is associated with satisfaction with existing institutions and reduced protest intentions, suggesting that neoliberal ideology helps stabilize unequal societies by justifying disparities, hence dampening resistance. The findings have urgent implications for Latin America and other contexts in which massive inequalities coexist with democratic institutions that depend on citizen engagement to function.

Haughton et al.' (2025) mixed‐methods analysis of UK media discourse on modern slavery provides a meticulous demonstration of how discursive power operates to maintain racial and national hierarchies while appearing to champion human rights. Analysing 2672 newspaper articles from 2013 to 2022, they reveal a fundamental contradiction in how modern slavery is represented. On one hand, media and political discourse position the United Kingdom as a moral leader in combating slavery, drawing on historical narratives about British abolition while ignoring the country's central role in the transatlantic slave trade. On the other hand, the same discourse criminalizes and delegitimizes the very people most likely to be slavery victims—migrants and asylum seekers, particularly those from Albania and other stigmatized nations.

This contradiction is not accidental but serves specific power functions. By portraying itself as fighting slavery abroad and protecting victims at home, the UK government claims moral authority on the global stage. Simultaneously, by questioning the authenticity of slavery claims by migrants and emphasizing abuse of the system, political discourse justifies harsh immigration policies and deportations. The dramatic acceleration of this anti‐immigration framing—with 73% of articles published in the last five years—coincides with Brexit and the implementation of the ‘hostile environment’ policy, revealing how discursive power intensifies during periods of political transformation.

The research demonstrates how power operates through what Foucault (1982) called ‘dividing practices’—techniques of power that categorize individuals and groups in ways that simultaneously individualize and totalize (see also Foucault, 1977)—creating categories of deserving and undeserving victims, legitimate and illegitimate claims, moral leaders and moral failures. These divisions are not neutral descriptions but performative constructions that create the realities they purport to describe. When the media repeatedly questions migrants' slavery claims, they create a climate of suspicion that deters actual victims from seeking help. When political discourse emphasizes British moral leadership, it obscures ongoing exploitation and deflects attention from structural reforms. The analysis reveals discursive power—wielded primarily by mass‐media outlets and their owners—as perhaps the most insidious form of domination, because it shapes the very terms through which people understand and contest reality.

These papers demonstrate how invisible power operates through hegemonic masculinity, social dominance orientation, authoritarianism, neoliberal ideology and media discourse to shape consciousness in ways that maintain hierarchies. But ideology does not operate in a vacuum—it is enacted through concrete intergroup relations and contested through collective action, our focus in Section 3.

### Section 3: Power in intergroup relations and collective action

The third section moves from examining how power shapes individual psychology and collective consciousness to investigating how it operates in dynamic intergroup relations and collective challenges to domination. These papers examine power not as possessed but as contested—negotiated through ongoing interactions that can reproduce or transform existing hierarchies.

Knab et al.' (2025) sophisticated analysis of intergroup helping reveals how power hierarchies can be maintained through seemingly benevolent actions. Their research across five studies in Germany and Israel (combined *N* = 1322) examines a paradoxical situation increasingly common in contemporary societies: majority group members simultaneously feel threatened by disadvantaged groups (refugees and Arab Israelis) while also perceiving strong social norms demanding that they help these groups. The resolution of this conflict, the authors demonstrate, often takes the form of dependency‐oriented helping that appears benevolent but actually maintains power differentials.

The key insight is that not all help is created equal from a power perspective. Autonomy‐oriented helping—providing resources, skills or opportunities that enable recipients to become self‐sufficient—can reduce power differentials by empowering recipients. Dependency‐oriented helping—providing temporary relief without addressing underlying inequalities or building capacity—maintains the helper's superior position while allowing them to feel virtuous. The research reveals a crucial interaction effect: when majority members perceive both high threat from disadvantaged groups and strong social norms to help, they specifically increase dependency‐oriented helping. This pattern was not observed for autonomy‐oriented helping, suggesting a targeted response that satisfies normative pressure while preserving hierarchies.

Particularly compelling is their longitudinal evidence (Study 2a) demonstrating that the interaction of norms and threat at one time point predicts dependency‐oriented helping eight weeks later, but not vice versa, suggesting causal direction. The research also reveals intriguing cross‐cultural variations: while the basic interaction held across contexts, in Germany, high threat combined with strong norms led to *increased* dependency‐oriented helping, whereas in Israel, strong norms only buffered against a general *decrease* in helping associated with high threat. This ‘wolf in sheep's clothing’ phenomenon (or ‘white saviorism’) reveals how power can be exercised through ostensibly positive behaviours, complicating simple distinctions between prejudice and benevolence.

Becker et al.' (2025) innovative use of virtual reality technology represents another methodological breakthrough that yields crucial theoretical insights. By immersing participants in a realistic protest scenario where they encounter police in either standard uniforms or full riot gear, the researchers can examine how visible displays of state power affect perceptions and behavioural intentions in ways impossible with traditional methods. The findings powerfully demonstrate that forceful displays of power can backfire: riot gear reduces perceived police legitimacy and increases resistance intentions, particularly among those initially less identified with the protest movement.

This research exemplifies how power is not simply imposed but dynamically negotiated through perceptions of legitimacy. According to the Elaborated Social Identity Model (Drury & Reicher, 1999, 2000, 2009) that guides this work, collective behaviour emerges from ongoing interactions between groups rather than being determined by either side alone. When police display excessive force readiness through a militarized appearance, they communicate that they view protesters as dangerous enemies rather than citizens exercising democratic rights. This delegitimizes police authority and can transform a heterogeneous crowd with diverse motivations into a unified oppositional force. The research has immediate practical implications for policing protests and theoretical implications for understanding how power operates through legitimacy rather than mere force.

Moon et al.' (2025) eight‐country study of collective action against organized crime exemplifies how structural contexts shape power dynamics and resistance. Their research examines an understudied form of power—that wielded by criminal organizations that operate as de facto authorities in many communities worldwide. From Italian mafias to Latin American cartels to Chinese triads, these groups exercise territorial control, extract resources, enforce their own ‘justice’ and shape cultural values in ways that blur the line between legitimate and illegitimate power.

The research tests two contrasting psychological pathways linking state responsiveness to citizen mobilization against organized crime. The ‘catalyst’ pathway suggests that responsive state institutions increase citizens' collective efficacy—their belief that community members can work together effectively—thereby promoting collective action. The ‘complacency’ pathway suggests that state responsiveness reduces perceived threat from criminal groups, thereby reducing motivation for citizen mobilization.

The findings support both pathways simultaneously, revealing the complex psychology of confronting alternative power structures. State responsiveness is associated with stronger collective efficacy, and this enhanced efficacy promotes a willingness to take action against criminal groups. But state responsiveness also reduces threat perceptions, which are linked to lower mobilization. While both pathways were significant, they work in opposite directions—state responsiveness both empowers communities through collective efficacy and reduces mobilization by diminishing threat perceptions. The positive catalyst effect outweighs the negative complacency effect, resulting in a modest net positive association with collective action intentions. This research emphasizes the need to examine these dynamics across various contexts: in countries where organized crime is deeply embedded, state responsiveness may matter more for collective efficacy, while in countries with a weaker criminal presence, the complacency effect could be stronger.

Jeong et al.' (2025) research with Black Americans offers a powerful counterpoint by examining how oppressed groups develop alternative conceptualizations of power that enable resistance despite structural disadvantages. Through two studies with over 600 Black U.S. American participants, they demonstrate that traditional social psychological definitions of power—as control over resources and influence over others—capture only a central *part* of how marginalized groups understand and mobilize power.

The research identifies five distinct forms of ingroup strength that Black Americans perceive as sources of power: collective resilience (the ability to persist and thrive despite adversity), ingroup solidarity (mutual support and unity), intergroup coalitions (alliances with other groups), ingroup resistance (capacity for protest and challenge) and intergroup respect (recognition from other groups). These alternative forms of power operate through different mechanisms than resource control. While traditional power predicts generalized perceptions of group influence, these ingroup strengths predict collective efficacy—the belief that the group can work together effectively to achieve goals—which in turn predicts organized resistance.

This reconceptualization has profound theoretical and practical implications. Theoretically, it challenges researchers to move beyond imposed definitions of power to understand how groups experiencing oppression conceptualize their own agency and capacity. It reveals that even groups labelled ‘powerless’ in traditional frameworks possess multiple forms of strength that enable them to challenge domination. Practically, it suggests that empowerment efforts should focus on strengthening group bonds, forming coalitions and developing resistance capacity as a means of attaining the resources and influence that comprise the most potent form of power.

The research also demonstrates the importance of recognizing everyday resistance alongside organized collective action. While social movements capture attention, marginalized groups also resist through daily acts of cultural preservation, mutual aid, consciousness raising and dignity maintenance. These forms of resistance may seem minor compared to organized action, but they sustain communities and preserve the capacity for mobilization when opportunities arise. By expanding the conceptualization of both power and resistance, this research offers a more complete understanding of how oppressed groups navigate and challenge systems of domination.

These papers reveal power as fundamentally relational and dynamic—constituted through helping relationships that can empower or disempower, through confrontations where authority displays can strengthen or undermine legitimacy, through state‐society relationships that affect collective efficacy, and through marginalized groups' development of alternative strengths. But all these dynamics unfold within particular cultural and historical contexts that fundamentally shape power's meaning and operation, leading to our final section.

### Section 4: Power across cultures and contexts

The fourth section directly confronts psychology's WEIRD problem by examining how power operates across diverse cultural contexts, revealing that many supposedly universal findings reflect particular cultural configurations rather than human universals.

Wasiel et al.' (2025) monumental 70‐culture study represents an excellent example of cross‐cultural research on power. Drawing on data from over 18,000 participants across all inhabited continents, they examine when and how position‐based power translates into social status. The massive scope allows them to test relationships very precisely while addressing cultural variation.

The research reveals three insights. First, the power‐status relationship varies dramatically across cultures, being strongest in societies characterized by high cultural embeddedness (where individuals are viewed as fundamentally embedded in social groups), high cultural tightness (with strong norms and low tolerance for deviance), low power distance (where perceptions are that society is more egalitarian than hierarchical) and low self‐expression values. It is weakest—sometimes approaching zero—in WEIRD societies and post‐Soviet contexts where egalitarian ideologies or historical experiences with authoritarian power have severed the assumed link between power and respect.

Second, individual‐level perceptions moderate these cultural effects. Across all cultures, perceiving powerholders as using their influence for collective benefit strengthens the power‐status link, while perceiving self‐serving use of power weakens it. This suggests a universal concern with how power is exercised, even as cultures vary in their baseline assumptions about power‐status relationships.

Third, the interaction between individual and cultural factors reveals that culture shapes not just mean levels but the very meaning of constructs. In some societies, power and status are so intertwined as to be nearly synonymous; in others, they represent distinct and potentially opposed hierarchies. This finding has profound implications for cross‐cultural collaboration, international business and global governance, where actors from different cultures may have fundamentally different assumptions about what power means and whether or under which conditions its holders deserve respect.

Kusano et al. (in press) theoretical analysis advances a fundamental rethinking of leadership in social psychology by addressing the conceptual fragmentation that has grown around some core constructs linked to the notion of power, including status, dominance and prestige. The influential dual‐pathway model casts dominance and prestige as distinct strategies for attaining leadership. The authors argue, instead, that such distinctions are often muddled by inconsistent definitions. They propose instead that leadership emerges through a single pathway of competence‐based status, socially conferred on individuals perceived as capable of addressing context‐specific collective needs. This reframing allows for a more precise understanding of why some traits, dominant or otherwise, translate into leadership in certain settings while failing in others.

The authors advance a socio‐ecological account that places followers' evaluations at the centre of leader selection. Leadership styles, they argue, are shaped by the match between the competencies a context demands and the traits a leader exhibits. Socio‐ecological conditions, such as resource scarcity, intergroup threat, economic stability or political safety, filter which competencies are most valued. In high‐threat environments, followers may prefer dominant leaders who can secure resources, enforce norms and protect against conflict. In peaceful contexts, non‐dominant styles such as servant or transformational leadership become more attractive, prioritizing cooperation, ethical integrity and mediation.

This account offers a flexible framework for understanding cross‐cultural and historical variation in leadership. Overall, the authors move beyond individualistic accounts of dominance and prestige to a model in which status through competence (as reflected in followers' needs and evaluation) is the universal foundation of leadership. This framework opens fertile ground for future research on cultural variation and the changing contextual demands that shape leadership over time and across spaces.

These papers decisively demonstrate that power cannot be understood through decontextualized universal laws but must be examined within the cultural meanings, historical experiences and structural contexts that shape its operation. They reveal variation so dramatic—from societies where power and status are nearly synonymous to those where they are uncorrelated—that any adequate theory must explicitly incorporate context rather than treating it as mere error variance. Together, the four sections point towards an understanding of power that integrates individual psychology, ideological processes, intergroup dynamics and cultural contexts—acknowledging their distinct logics while examining their mutual constitution.

## THEORETICAL INTEGRATION: SIX FUNDAMENTAL SHIFTS IN UNDERSTANDING POWER

The diverse contributions to this special issue, while examining different aspects of power across different contexts, converge on several fundamental insights that collectively redefine how social psychology should approach power. These insights not only challenge existing paradigms but also point towards new theoretical frameworks, methodological approaches and practical applications.

### From unitary to plural conceptions

Perhaps the most fundamental shift represented in this special issue concerns not what power is but where power is located and how it operates. We recognize that the papers in this issue share a basic understanding that power involves resources, influence and control. The crucial advance lies in examining the multiple sites, forms and mechanisms through which such control operates—and how these can diverge, align or transform depending on context.

Traditional approaches locate power primarily in individual possession of resources that enable influence over others. This remains important, as the Bartholomaeus et al. (2025) and Wildschut and Insko (2025) papers demonstrate. However, this special issue reveals at least four additional sites where power operates:

#### Power as structural position

Several papers examine how power is embedded in group memberships, institutional roles and historical legacies rather than individual attributes. Wasiel et al.'s (2025) 70‐culture study reveals that whether holding formal authority translates into social status depends entirely on cultural context—in some societies, the relationship approaches zero. Moon et al. (2025) examine how state responsiveness shapes communities' capacity to resist criminal organizations. These papers locate power not in what individuals possess but in the structural positions they occupy and whether those positions command respect, resources or legitimacy.

#### Power as relational process

Other papers examine power as contested through ongoing interaction rather than possessed as a static attribute. Becker et al.'s (2025) virtual reality research demonstrates that police displays of force can strengthen or undermine their authority depending on how protesters interpret the display—power is constituted through the relationship, not unilaterally imposed. Knab et al. (2025) show how helping relationships create or maintain hierarchies depending on whether help fosters autonomy or dependency. Here, power exists not in either party but in the dynamic between them.

#### Power as discursive construction

Still other papers locate power in control over meaning‐making, narrative framing and collective consciousness. Haughton et al. (2025) demonstrate how media discourse about modern slavery simultaneously positions the United Kingdom as a moral leader while criminalizing the very migrants most likely to be victims—power operates through constructing categories of deserving/undeserving that shape policy and public opinion. Avery et al. (2025) show how hegemonic masculinity and ecological dominance operate as ideological frameworks that make environmental protection appear threatening to those invested in patriarchal hierarchies. Solano‐Silva et al. reveal how neoliberal ideology makes objectively unjust inequalities appear fair. In these cases, power operates not through direct resource control but through shaping the interpretive frameworks through which people make sense of reality.

#### Power as collective capacity

Finally, several papers locate power not in what individuals or even groups possess but in their collective capacity for coordinated action. Jeong et al. (2025) identify collective resilience, solidarity and resistance capacity as forms of strength that enable marginalized groups to challenge domination despite lacking traditional resources. Moon et al. (2025) examine collective efficacy—communities' belief in their ability to work together—as crucial for mobilizing against organized crime. These papers recognize that power can emerge from mutual constitution (Shweder & Sullivan, 1993)—the ways individuals, groups and contexts dynamically shape each other—creating capacities that exist only at the collective level.

Understanding power requires examining all these sites and how they interact. Traditional resource control remains important but operates differently depending on structural positions, relational dynamics, discursive framings and collective capacities. A wealthy individual has visible power through resources, but their hidden power (agenda‐setting) and invisible power (shaping consciousness) depend on occupying positions that command authority, participating in relationships that are seen as legitimate, controlling meaning‐making institutions and potentially facing organized collective resistance. These different forms can align, as when economic dominance translates into political influence and ideological hegemony. But they can also diverge, as when groups with few traditional resources develop alternative strengths that enable successful resistance, or when authorities' displays of force backfire by undermining legitimacy.

This multi‐sited understanding helps resolve apparent tensions between papers using traditional power manipulations and those examining alternative conceptualizations. Both examine power, but at different sites and through different mechanisms. The former focuses on visible power as individual resource control; the latter examines invisible power operating through consciousness or collective capacity emerging from solidarity. The diversity reflects power's genuine complexity rather than conceptual confusion.

Moreover, these various forms of power exist in complex relationships of mutual constitution, competition and transformation. Economic power can be converted into political influence, which shapes cultural narratives, which influence psychological states, which motivate or suppress collective action, challenging or supporting the political‐economic status quo. Understanding power requires theories that can accommodate this plurality while identifying patterns across different manifestations. The special issue points towards ecological or complex‐systems approaches that examine how different forms of power interact within specific contexts rather than seeking universal laws that apply regardless of form or setting.

### From static to dynamic perspectives

The research in this special issue decisively moves beyond treating power as a static attribute of individuals or groups to examining it as a dynamic, performative and contested phenomenon. Power is not simply possessed but must be continually enacted and defended. It exists not in isolation but in relationships that evolve through interaction. Attempts to assert power can strengthen or undermine it depending on how they are received and resisted. Power relations that seem stable can rapidly transform when conditions change or consciousness shifts.

This dynamic perspective requires new theoretical tools that can capture emergence, feedback loops, tipping points and transformations. It suggests studying power longitudinally and processually rather than through cross‐sectional snapshots. It demands attention to the mechanisms through which power relations are reproduced or disrupted over time. The virtual reality protest studies exemplify this approach by examining how power displays dynamically shape legitimacy perceptions and resistance intentions through unfolding interactions.

### From individual to multilevel analysis

While traditional social psychology has focused primarily on individual‐level power dynamics, this special issue demonstrates that power operates simultaneously across multiple levels that mutually influence each other. Individual experiences of empowerment or disempowerment cannot be understood outside the group dynamics, cultural meanings and structural positions that shape them. Conversely, macro‐level power structures are enacted and potentially transformed through individual and collective psychology.

The papers model various approaches to multilevel analysis. Some examine cross‐level interactions, showing how individual‐level processes like authoritarianism interact with country‐level factors like religiosity. Others trace how cultural‐level ideologies like neoliberalism shape individual‐level perceptions and behaviours. Still others demonstrate how group‐level processes like collective efficacy mediate between structural conditions and individual actions. Together, they point towards integrated multilevel theories that can capture power's operation across scales while respecting the distinct dynamics at each level.

### From universal to contextual understanding

Few themes stand out more clearly than the central role of cultural and historical context in shaping our understanding of power. The assumption that findings from WEIRD populations represent human universals has been thoroughly discredited. Power's very meaning—whether it implies responsibility or opportunity, whether it correlates with status, whether it requires force or knowledge—varies dramatically across societies. Historical legacies of colonialism, slavery, dictatorship and revolution create enduring templates that shape contemporary power relations. Current ideological contexts like neoliberalism or authoritarianism influence how people perceive and enact power.

This contextual understanding does not imply pure relativism—the papers identify patterns that hold across contexts, such as concerns about self‐serving uses of power. But it does require theories that explicitly incorporate context rather than merely treating it as error variance. It demands a historically informed analysis that examines how past power relations shaped and influenced present conditions. It necessitates collaborative research with scholars from diverse contexts rather than imposing frameworks developed in Western academia. The 70‐culture study and Latin American justice research exemplify how centring context can reveal patterns invisible from any single cultural vantage point.

### From domination to resistance

Traditional power research has often focused on how those with power use it to influence, control or exploit others. While this remains important, the special issue dramatically expands the focus to examine how those subjected to power understand, navigate and resist it. The papers reveal that even groups considered ‘powerless’ in traditional frameworks possess multiple forms of agency and strength that enable them to challenge domination.

This shift requires theoretical frameworks that can accommodate both domination and resistance as interconnected processes. It means studying not just how power operates but how it fails, not just how hierarchies are maintained but how they are challenged, and not just successful influence but active resistance. The research on Black Americans' alternative conceptualizations of power and everyday resistance exemplifies this approach, showing how marginalized groups develop sophisticated strategies for preserving dignity and creating change despite structural disadvantages.

### From description to transformation

Finally, the special issue moves beyond merely describing power dynamics to examining how they can be transformed. Several papers directly test interventions or identify mechanisms that could promote more equitable power relations. The power construal research shows that changing how people think about power can change how they use it. The protest policing studies demonstrate that less forceful displays of authority can be more effective at maintaining order. The collective efficacy research identifies psychological resources that enable communities to challenge and shake off their own domination.

This transformative orientation reflects growing recognition that psychological science should not simply document inequalities but contribute to addressing them. It requires research designs that test not just whether interventions work but how and why, attention to unintended consequences and backfire effects, and partnership with communities working for change. The special issue models how rigorous science can serve social justice by providing evidence about how power operates and how it might operate differently.

## IMPLICATIONS FOR RESEARCH AND PRACTICE

The theoretical advances represented in this special issue have profound implications for how future research should approach power and how practitioners should address power‐related challenges.

### Methodological implications

The special issue demonstrates the need for methodological pluralism in power research. While laboratory experiments remain valuable for establishing causal relationships, they must be complemented by methods that capture power's complexity in naturalistic settings. The papers employ an impressive range of methods—cross‐cultural surveys, daily diaries, virtual reality, discourse analysis, psychometric development—each suited to different research questions. Future research should continue this methodological innovation while developing new approaches suited to revealing power's multilevel, dynamic nature.

Several specific methodological needs emerge from this special issue. First, we need longitudinal and processual methods that capture how power relations unfold and transform over time, rather than static snapshots. The dynamic nature of power revealed across these papers—from Becker et al.'s (2025) demonstration that authority displays can backfire to Moon et al.'s (2025) examination of how state responsiveness affects collective efficacy over time—demands methods that can capture emergence, feedback loops and transformation. Second, we need mixed methods that combine quantitative tests of relationships with qualitative insights into meaning‐making and lived experience. Haughton et al.'s (2025) discourse analysis and Jeong et al.'s (2025) exploration of alternative power conceptualizations demonstrate how qualitative approaches can reveal dimensions of power invisible to quantitative measures alone, while quantitative cross‐cultural research like Wasiel et al.'s (2025) can test patterns across contexts at scale. Integrating both approaches within single research programmes can provide complementary insights. Third, we need participatory approaches that engage affected communities as partners rather than subjects, recognizing their expertise about power dynamics born from lived experience. As liberation psychology emphasizes, those experiencing oppression possess crucial knowledge often invisible from positions of privilege (Martín‐Baró, 1994). Fourth, we need cross‐cultural collaboration that ensures research questions, methods and interpretations reflect diverse perspectives rather than imposing Western frameworks. The dramatic cross‐cultural variation revealed in this issue demonstrates that power cannot be understood through WEIRD samples alone. Finally, we need ecological momentary assessment and other intensive longitudinal methods that capture how power operates in daily life across contexts, as exemplified by Farkhari et al.'s (2025) daily diary approach.

### Theoretical implications

The special issue points towards several crucial theoretical developments. First, we need integrative frameworks that can accommodate power's plurality—its operation through multiple forms and mechanisms—while identifying general principles across manifestations. The multi‐dimensional framework we outlined earlier, distinguishing visible, hidden and invisible power, provides one possible foundation, but we need theories that specify relationships among dimensions and predict when they will align or diverge. Second, we need process theories that explain how power relations emerge, stabilize, transform and collapse over time. Static models treating power as an attribute cannot explain the dynamic phenomena revealed across these papers—how authority can suddenly lose legitimacy, how consciousness can shift to enable mobilization, how alternative power bases can be built. Third, we need multilevel theories that specify relationships between individual, interpersonal, group, institutional and cultural levels of power. Many papers in this issue implicitly operate across levels, but we lack comprehensive theories that explain cross‐level influences and emergence. Fourth, we need contextual theories that explicitly incorporate cultural meanings and historical legacies rather than seeking decontextualized universals. The dramatic variation across contexts demands theories that treat context not as error but as constitutive. Fifth, we need critical theories that examine not just how power operates but whose interests it serves and how it might be transformed. Liberation psychology provides one model; feminist, decolonial and other critical traditions offer additional resources. Finally, we need intersectional theories that examine how different forms of power and oppression combine rather than treating them separately, recognizing that people occupy multiple positions simultaneously that cannot be understood in isolation.

We would be remiss in failing to note the absence of papers focusing on class, as well as papers from the liberation psychology tradition. The continued dominance of the United States within psychology is evident here: its political culture, in which class is anathema and invisibilized, reveals its influence. The two most potent forms or modalities of power— economic and military— which liberation psychology seeks means of overcoming, and which tenured psychology professors in Global North countries rarely feel the sharp end of, are unexamined here. The political economy of academia— those political and economic pressures operating in universities that influence knowledge production— leaves a telltale mark in the form of which topics are left untouched.

However, the special issue's emphasis on structural positions and cultural meanings resonates strongly with recent theorizing about social class as context (Kraus et al., 2012; Stephens et al., 2014). This framework recognizes that socioeconomic position shapes not just access to resources but fundamental aspects of psychological experience—cognition, emotion, motivation, behaviour and even neural processes (Manstead, 2018). Importantly, class operates not as a simple individual attribute but as a contextual factor that structures the environments people navigate, the opportunities and constraints they face and the cultural models available for making sense of experience.

Several parallels between the class‐as‐context literature and this special issue's contributions warrant attention. First, both recognize that structural position profoundly shapes psychological experience in ways often invisible to those occupying privileged positions. Just as Kraus et al. (2012) demonstrate that higher social class is associated with increased self‐focus and reduced social engagement, Wasiel et al.'s (2025) cross‐cultural research reveals that whether formal authority commands respect depends entirely on cultural context—power's psychological meaning cannot be understood apart from the structural and cultural contexts in which it operates.

Second, both literatures emphasize cultural variation in how structural position translates into psychological outcomes. Miyamoto et al. (2018) show that culture moderates the psychological effects of socioeconomic status, with social class predicting different patterns of cognition and behaviour in the United States versus Japan. Similarly, Gobel et al.'s (2025) cross‐cultural research in this issue reveals that how people construe power—as achieved through merit or entrusted by others—varies across cultures and fundamentally shapes whether power promotes prosocial or antisocial behaviour. In both cases, the lesson is that structural positions (whether class or power) do not have universal psychological effects but operate through culturally shaped meanings and practices.

Third, both recognize the need to examine multiple dimensions of stratification simultaneously. Just as social class researchers increasingly recognize that class intersects with race, gender and other dimensions of inequality (Crenshaw, 1989; Ridgeway, 2014), this special issue demonstrates that power cannot be understood in isolation from the ideological frameworks (Avery et al., 2025 on hegemonic masculinity), historical legacies (Haughton et al., 2025 on colonial narratives) and collective capacities (Jeong et al., 2025 on alternative strengths) that shape its operation.

However, the special issue also extends beyond existing class literature in important ways. While class research typically focuses on socioeconomic gradients within societies, many papers here examine power relations across societies and cultural contexts, revealing far greater variability in power's meaning and operation than within‐society analyses typically capture. Additionally, the special issue's emphasis on resistance and transformation—how marginalized groups develop alternative conceptualizations of power and mobilize for change—complements the class literature's focus on how stratification shapes individual psychology by examining collective processes of empowerment and social change.

These connections suggest fruitful directions for future research. How do class‐based power differentials interact with other forms of power examined in this issue—ideological hegemony, discursive control, collective capacity? How do cultural contexts shape not just the psychological consequences of occupying different class positions but the very meaning of class itself? And how might insights from liberation psychology about collective empowerment inform interventions to address class‐based inequalities? By building bridges between power research and the class‐as‐context literature, we can develop more comprehensive understandings of how structural positions shape psychology while remaining attentive to resistance and transformation.

### Practical implications

For practitioners working to address power‐related challenges—whether in organizations, communities, or societies—the special issue offers crucial insights. First, attend to construal: How people understand power shapes its effects. Gobel et al.'s (2025) research demonstrates that reminding powerholders their authority was entrusted by others promotes prosocial behaviour, suggesting interventions can shift construal to promote more responsible power use. Second, recognize multiple forms: Empowerment requires recognizing and building diverse forms of power beyond resource transfer. Jeong et al.'s (2025) identification of collective resilience, solidarity and resistance capacity as sources of strength suggests that empowerment efforts should focus on building group bonds and collective capacities, not just transferring material resources—though such capacity‐building may be the most effective route to eventually obtaining traditional resources. Third, consider system dynamics: Interventions in complex systems of power can produce unexpected results. Becker et al.'s (2025) finding that heavy‐handed displays of authority may backfire by undermining legitimacy demonstrates the need to anticipate how power assertions will be interpreted and potentially resisted. Fourth, address multiple levels: Lasting change requires addressing individual attitudes, group dynamics, institutional structures and cultural meanings simultaneously. Changing one level while leaving others intact is unlikely to produce sustainable transformation. Fifth, partner with communities: Those experiencing power dynamics have crucial expertise born from lived experience. Effective interventions emerge from partnership rather than imposition, as liberation psychology has long emphasized. Finally, examine intersections: Power operates through multiple, intersecting systems of advantage and disadvantage—based on class, race, gender, nationality and more—that must be addressed together rather than in isolation. Interventions targeting one dimension while ignoring others may fail or produce unintended consequences.

## CONCLUSION: TOWARDS A NEW PSYCHOLOGY OF POWER

This special issue marks an important step forward in the social psychology of power. By expanding conceptualizations, embracing methodological innovation, centring cultural diversity and integrating multiple levels of analysis, it provides foundations for a more comprehensive and nuanced understanding of power in human societies. The collected papers demonstrate that power is not a simple force of domination but a complex, multifaceted, culturally embedded and dynamically contested phenomenon that shapes every aspect of human social life.

The implications extend far beyond academic psychology. In an era of rising authoritarianism, persistent racism, ecological crisis, global upheaval and the pervasive influence of various criminal systems, understanding power's psychological dimensions becomes ever more crucial. How can societies resist the appeal of strongman politics? How can movements for racial, gender and economic justice build power to challenge entrenched hierarchies? How can humanity cooperate to address existential threats that require coordinating across vast differences in power? These questions demand the kind of sophisticated, contextual, multilevel understanding of power that this special issue advances.

Perhaps most importantly, the research collected here demonstrates that power is not the fixed possession of the few but a dynamic relationship that can be transformed. Even groups marginalized by traditional hierarchies possess forms of strength that enable them to resist and create change. Power's effects depend on how it is understood and enacted, opening possibilities for interventions that promote more prosocial uses. Seemingly stable hierarchies can be disrupted when legitimacy erodes or consciousness shifts. By expanding our vision of what power is and how it operates, we expand possibilities for human agency and social transformation.

The journey to redefine power in social psychology has only just begun. This special issue marks not an endpoint but a departure point for new questions, methods and possibilities. As power dynamics continue to evolve in our rapidly changing world, so too must our efforts to understand and shape them. The future of power research lies in continued innovation, cultural humility and commitment to using psychological science in the service of human flourishing and social justice. The papers collected here light the way forward, demonstrating that a psychology of power adequate to our times must be as complex, dynamic and diverse as power itself.

## AUTHOR CONTRIBUTIONS

**Karim Bettache:** Conceptualization; writing – original draft; writing – review and editing. **Giovanni A. Travaglino:** Writing – review and editing. **Peter Beattie:** Writing – review and editing.

## FUNDING INFORMATION

This work was supported by the UKRI under Grant ‘Secret Power’ No. EP/X02170X/1 (awarded to GA Travaglino under the European Commission's ‘European Research Council – STG’ Scheme).

## Data Availability

Data sharing not applicable to this article as no datasets were generated or analysed during the current study.
